# Correction: Nocini et al. Improving Nasal Protection for Preventing SARS-CoV-2 Infection. *Biomedicines* 2022, *10*, 2966

**DOI:** 10.3390/biomedicines13071698

**Published:** 2025-07-11

**Authors:** Riccardo Nocini, Brandon Michael Henry, Camilla Mattiuzzi, Giuseppe Lippi

**Affiliations:** 1 Unit of Otorhinolaryngology, Department of Surgery, Dentistry, Paediatrics and Gynaecology, University of Verona, Piazzale L.A. Scuro 10, 37134 Verona, Italy; 2 Division of Nephrology and Hypertension, Cincinnati Children’s Hospital Medical Center, 3333 Burnet Ave, Cincinnati, OH 45229, USA; 3 Service of Clinical Governance, Provincial Agency for Social and Sanitary Services (APSS), Via Alcide Degasperi 79, 38123 Trento, Italy; 4 Section of Clinical Biochemistry and School of Medicine, University of Verona, Piazzale L.A. Scuro 10, 37134 Verona, Italy

## Correction in the Main Text

In the original publication [1], a correction is needed in the first paragraph in Section 3.2.2 as published. Part of the text related to reference 52 of Balmforth et al. (https://pubmed.ncbi.nlm.nih.gov/40374451/) needs to be removed, since the article has been retracted after the publication of the present article. A correction has been made to Section 3.2.2. With this correction, the order of some references has been adjusted accordingly.

3.2.2. Human Clinical Studies

Evidence emerged by the study of Figueroa and colleagues, using an IC-containing nasal spray, which was randomly administered with placebo to 394 Hospital workers [52]. All study subjects were asked to self-administer 1 puff (0.10 mL, 0.17 mg of IC or placebo) four times daily in both nostrils. The number of diagnoses of SARS-CoV-2 infection after 21 days of treatment was found to be significantly lower in subjects who used the IC nasal spray (2/196) compared to those who used the placebo (10/198; absolute risk reduction, 4%; 95%CI, 0.6–7.4%). No side-effects analysis was carried out in this investigation. 

Paolacci et al. administered a nasal spray containing α-cyclodextrin and hydroxytyrosol to 149 healthy volunteers at high risk of SARS-CoV-2 infection due to their occupation [53]. During 30 days of follow-up, none of these subjects was diagnosed with COVID-19, although no SARS-CoV-2 infection also occurred in 76 control individuals who did not use the nasal spray during the same period. No side-effects analysis was carried out in this work.

Preliminary but interesting evidence has also been provided that an anti-SARS-CoV-2 neutralizing monoclonal antibodies (mAbs)-based nasal spray may provide good protection against SARS-CoV-2 infection. In a small-scale clinical trial, Lin et al. explored the efficacy of a single nasal spray containing the anti-SARS-CoV-2 35B5 mAb, which was tested in 30 healthy volunteers [54]. It was found that the mAB 35B5 concentration in a nasal mucosal specimens remained significant up to 72 h after spray administration (1 mg/mL 35B5 mAb diluted in 50% Dulbecco’s phosphate-buffered saline with 50% glycerol), concomitantly conveying up to 24–48 h efficient in vitro neutralization of several SARS-CoV-2 variants of concern (VOCs), including Omicron.

Recent evidence has been provided that the anticoagulant drug heparin displays pleiotropic antiviral properties, mostly by binding to the spike protein of SARS-CoV-2 and thus inhibiting host cell infection [55]. It is hence not surprising that its administration through the unconventional nasal route has been conceived as a prophylactic treatment against SARS-CoV-2 infection [56]. To this end, Eder et al., carried out a single-center, open-label intervention study aimed at exploring the effect of low molecular weight heparin (LMWH) inhalation for preventing SARS-CoV-2 infection [57]. By means of a nebulizer, 33 subjects received 4500 IU Enoxaparin or placebo in the right or left nostrils, respectively. Nasal epithelial cells were then collected by brushing and exposed to both authentic SARS-CoV-2 isolate and SARS-CoV-2 pseudovirus. In both cases binding to, and infection of, human nasal cells were found to be substantially inhibited by enoxaparin for up to 16 h.

The study protocol of a phase III, double-blind, randomized, single-centre clinical trial for establishing the efficacy of carrageenan-based nasal spray (purified water, 0.5% sodium chloride, 1.2 mg iota-carrageenan and 0.4 mg kappa-carrageenan) for lowering the risk of SARS-CoV-2 infection was presented in September 2022 [58], but results are still unavailable.

The authors state that the scientific conclusions are almost unaffected. This correction was approved by the Academic Editor. The original publication has also been updated.
